# Prevalence of genetic mutations in alpha-1 antitrypsin deficiency (aatd) in patients with chronic obstructive pulmonary disease in Colombia

**DOI:** 10.1186/s12890-023-02453-0

**Published:** 2023-05-04

**Authors:** Abraham Alí-Munive, Prada Leidy, Nadia Juliana Proaños, John Pedrozo-Pupo, Angela Giraldo, Diana Cano, Claudia Diaz-Bossa, Ricardo Mosquera, Hector Paul, Mauricio Gonzalez-García, Carlos Aguirre-Franco, José Luis López-Campos, Alejandro Casas-Herrera

**Affiliations:** 1 Fundación Neumológica Colombiana, Cra. 13 B # 161 – 85, Bogota, Colombia; 2 Universidad de La Sabana, Campus del puente del común, Km 7 Autopista Norte de Bogotá, Chía, Chía- Cundinamarca, Colombia; 3 Respire Instituto para el Cuidado Respiratorio (Previcare Ltda), Cra 21 # 18 – 27., Santa Marta, Colombia; 4 CardioSalud Eje Cafetero SAS, Cra. 13 # 3B ? 12, Pereira, Colombia; 5 Instituto Neumologico del Oriente S.A. Calle, 53 No. 31 – 30. Bucaramanga, Santander, Spain; 6 Neumomed SAS, Calle 19 A No. 44 ? 25, Medellin, Antioquia, Colombia; 7 Clínica Neumológica del Pacífico S.A.S. Calle 5 A No. 42A – 18. Cali, Valle, Barranquilla, Colombia; 8 Promotores de la salud de la costa S.A.S. Cra 54 No 54 ? 01, Barranquilla, Colombia; 9 Unidad Médico-Quirúrgica de Enfermedades Respiratorias, Hospital Universitario Virgen del Rocío, Avda de Manuel Siurot s/n, Sevilla, 41013 España

**Keywords:** COPD, Pulmonary emphysema, alpha-1 antitrypsin deficiency, Genetic mutation, Genotyping test

## Abstract

**Background:**

Alpha-1 antitrypsin deficiency (AATD) is an underrecognized genetic disorder associated mainly with pulmonary emphysema and Chronic Obstructive Pulmonary Disease (COPD). All individuals with COPD regardless of age or ethnicity should be tested for AATD, but in Colombia its prevalence in unknown.

**Main objective:**

To determine the prevalence of the genetic mutations, present in AATD in adult patients with COPD in Colombia, using a genotyping test on cells from the oral mucosa.

**Methods:**

This was a multicentre, observational, cross-sectional study which included adult patients attending seven COPD care centres in Colombia. Demographic data, medical history, including history of exposure to smoking and biomass smoke, most recent spirometry, pharmacological and non-pharmacological treatment received, serum AAT levels, and mutations detected by the genotyping test were recorded for all the recruited patients. For the comparison of variables between the groups with and without mutation, we used the X^2^ test for the qualitative variables and the Student’s t-test or Mann-Whitney U test according to their distribution.

**Main findings:**

We collected a sample of 1,107 patients, the median age was 73.8 years (87.6–79.9). Mutations were documented in 144 patients (13.01%), the majority had the M/S mutation (78.50%), followed by M/Z (9.72%). One patient had a ZZ mutation and two patients had null alleles. In total, 23 patients had mutations associated with serum AAT deficiency (levels below 60 mg/dl).

**Conclusions:**

Genetic mutations were documented in 13.01% of patients with COPD in Colombia and 2.07% were AATD-related, showing that there is a significant number of underdiagnosed patients.

## Introduction

Alpha-1 antitrypsin deficiency (AATD) is a hereditary condition associated with a higher risk of developing lung disease, in the form of emphysema, and liver disorders, depending on the allelic variants expressed [1]. Currently, the measurement of alpha-1 antitrypsin levels is recommended in all patients with chronic obstructive pulmonary disease (COPD) regardless of age and severity of the disease [2]. However, this test is rarely requested, and no studies have been carried out in Colombia to determine the prevalence of the disease. In Colombia, there seems to be the perception that the population does not suffer from this genetic condition and its prevalence in the COPD patient population is therefore unknown.

Reviewing the epidemiology, the prevalence of AATD in the general population is reported to be from 1 to 2,000 to 1 in 5,000 individuals in some parts of Europe, and from 1 to 5,000 to 1 in 10,000 in the United States and Canada, while these figures are much lower in Latin American countries [3–6]. Analysing patients with COPD only, the prevalence of AATD is 10%, including all mutations. The prevalence of severe AAT deficiency has been estimated at 1–2% [3, 4, 6–9].

Although there have been previous estimates of the prevalence of AATD in Colombia, no studies have been conducted to confirm the figures. The aims of this study were to determine the prevalence of the genetic mutations present in AATD in adult patients with COPD in Colombia using a genotyping test on cells from the oral mucosa and to describe the general characteristics of the subjects with genetic mutations. An exploratory objective was to evaluate differences in comorbidities, and lung function in subjects with and without mutations.

## Methodology

This was a multicentre, observational, cross-sectional study, which included patients attending seven COPD care centres in the North (Barranquilla, Santa Marta), Centre (Medellín, Bogotá, Pereira, Bucaramanga) and South (Cali) of Colombia.

### Patients

We included adult patients with a diagnosis of COPD invited to take part at the specialised care clinics at the chosen centres from 25 to 2020 to 31 January 2022. The inclusion criteria were: being over 18 years of age; previous diagnosis of COPD (post-BD spirometry with FEV1/FVC < 70%) with a spirometry report from within the previous two years; no previous study to measure AAT levels; no previous AAT genotyping study; and provision of written informed consent for participation in the study. Patients with symptoms of lower or upper respiratory tract infection, at least during the four weeks prior to inclusion, and any who had received antibiotics or systemic corticosteroids for the treatment of respiratory exacerbation in the four weeks prior to inclusion were all excluded. Subjects were enrolled in the study using a non-random sampling technique of consecutive cases.

Demographic data, medical history, including history of exposure to smoking and biomass smoke as risk factors for COPD, number of previous exacerbations, most recent spirometry, scores for comprehensive assessment of the disease such as the BODEx index and CAT score, comorbidity indices, pharmacological and non-pharmacological treatment received, serum AAT levels, and mutations detected by the genotyping test were recorded for all the recruited patients.

### Sample size

The sample size was calculated using version 13 of the STATA program with the following parameters: estimated prevalence 13%; confidence level 95%; and precision 2%. With these parameters, a sample size of 1,015 subjects was obtained [9, 10].

### Sample handling

Samples were taken at the institutions of origin by means of oral swabs, with the Oracollect OCR-100 device, at no cost to the participants, and sent to Spain for analysis at central laboratories (Progenika Biopharma, Vizcaya, Spain) [11]. The device is intended for use in the non-invasive collection of oral mucosa samples. Human DNA from the oral mucosa sample is isolated and stabilised and is suitable for use in molecular diagnosis applications. Oral mucosa samples are stabilised in the internal fluid and can be transported and stored under ambient conditions. The device contains a bacteriostatic stabilising fluid that inhibits the growth of bacteria from sample collection until processing. The samples were registered on the internet Platform (https://grifolsalpha1test.com/) with a unique code individually associated with each sample sent.

### Genetic assessment

To identify the allelic variants in the patients included in the study, molecular analysis of the SERPINA1 gene was performed. The genotyping was performed by direct genotyping with the A1AT Genotyping Test diagnostic kit (Progenika Biopharma, Vizcaya, Spain), which simultaneously analyses the 14 most prevalent mutations in the world associated with the disease from DNA extracted from a sample of oral mucosa. This test uses polymerase chain reaction (PCR) amplification to obtain large numbers of target sequences in the alpha-1 antitrypsin gene, SERPINA1. The PCR products are hybridised with fluorescent labelled probes that are detected with a Luminex® 200 system [11].

### Data collection

Before starting recruitment, each center was trained in sample handling, eligibility criteria and the use of data collection forms. All the study information was collected in the electronic data capture software (REDCap) by the main investigator and co-investigators. REDCap allowed minimization of typing errors and preserved the confidentiality of the subjects.

### Statistical analysis

Prior to the statistical analysis, it was verified that the recorded data corresponded to the type of variable, its corresponding unit, and the coding, if applicable. The qualitative variables are described in absolute frequencies and percentages, and the quantitative variables in measures of central tendency and dispersion according to the assumption of normality evaluated by the Kolmogorov-Smirnov test. For the comparison of variables between the groups with and without mutation, we used the X2 test for the qualitative variables and the Student’s t-test or Mann-Whitney U test according to their distribution. The statistical software STATA 13 was used for data analysis.

### Ethical considerations

The study was approved by the research ethics committee of the Colombian Respiratory Medicine Foundation (Fundación Neumológica Colombiana), as stated in minute 257 of 18 August 2020. This research was carried out in accordance with the guideline for Good Clinical Practice, under the guidelines established in the Declaration of Helsinki and according to resolution No. 008430 of 1993, which sets out the scientific, technical and administrative standards for health research in Colombia.

The patients authorised their participation in the study by giving their written informed consent. Participant confidentiality was maintained by replacing their names with numerical codes assigned solely for the purposes of the study. The database was anonymised and there were no data that might enable patient identification, to guarantee their confidentiality. Additionally, biological samples were rendered anonymous with a consecutive code.

## Results

During the study period, we collected a sample of 1,107 patients, 62.1% of whom were male; the median age was 73.8 years (67.6–79.9) and the median age at onset of symptoms was over 60. Mutations were documented in 144 patients (13.01%).

When analysing the differences between patients with mutations and those with no mutations, we found no clinical features indicating any differences when comparing by gender, age, body weight, body mass index, age at onset of symptoms, age at diagnosis, exacerbations of all types, and frequency of comorbidities, including smoking and exposure to biomass (see Table 1).

**Table 1:** General characteristics of the population

Variables	No mutation n = 963	Mutation n = 144	Total n = 1,107	p-value
Male, n (%)	602 (62.1)	91 (63.2)	693 (62.6)	0.875
Age, median (p25-p75)	73.8 (67.6–79.9)	73 (66.6–79.9)	73.8 (67.4–79.9)	0.428
Weight, median (p25-p75)	65 (56–74)	65 (56.5–75)	65 (56–74)	0.468
BMI, median (p25-p75)	25.1 (22.1–28.7)	25.2 (22.8–28.5)	25.1 (22.2–28.6)	0.410
Age at onset of symptoms, median (p25-p75)	64 (55–70)	63 (55–70)	64 (55–70)	0.971
Age at diagnosis, median (p25-p75)	65 (58–72)	65 (59–72)	65 (58–72)	0.972
Moderate exacerbations in the previous year, mean ± SD	0.25 ± 0.65	0.20 ± 0.66	0.24 ± 0.65	0.424
Severe exacerbations in the previous year, mean ± SD	0.09 ± 0.35	0.11 ± 0.28	0.10 ± 0.35	0.514
**Comorbidities and exposure factors**				
HT, n (%)	521 (54.1)	78 (54.2)	599 (54.1)	0.988
Diabetes mellitus, n (%)	119 (13.4)	21 (14.6)	140 (12.6)	0.453
Heart failure, n (%)	169 (17.5)	18 (13.5)	187 (16.9)	0.131
Smoking, n (%)	691 (71.7)	108 (75)	799 (72.2)	0.418
Years smoking, median (p25-p75)	38 (24–47)	35 (20–45)	38 (22–47)	0.283
PY, median (p25-p75)	30 (15–50)	25 (10-49.5)	30 (15–50)	0.119
s-hand smoke, n (%)	189 (19.6)	25 (17.4)	214 (19.3)	0.517
Biomass, n (%)	297 (30.8)	34 (23.6)	331 (29.9)	0.077
Biomass index, median (p25-p75)	90 (40–200)	85 (60–160)	90 (40–200)	0.858
Tuberculosis, n (%)	51 (5.3)	12 (8.33)	63 (5.7)	0.142
Occupational smoke, n (%)	117 (13.6)	15 (10.9)	132 (13.2)	0.386

BMI: body mass index; HT: hypertension, PY: pack-years, P: differences between subjects with and without the mutation. Values as mean ± standard deviation or median (p25-p75) or n (%)

We compared the non-pharmacological treatment received by the patients in each group, with and without mutations. When analysing vaccination against influenza, pneumococcus and pulmonary rehabilitation, the groups were the same, except for oxygen therapy, which showed a statistical difference in favour of the group with mutations; 45.5% (p = 0.004). Most of the patients had baseline bronchodilator treatment with dual bronchodilator therapy followed by triple therapy, the figures being similar for patients with and without genetic variations.

The available data included spirometry, to assess the patients’ lung function at the time of recruitment. The FEV1/FVC ratio was close to 55% in both groups, with and without mutations, with no statistically significant difference. There were also no differences found with the post-bronchodilator FEV1, with a value of 1.22 L, nor in the distribution of groups according to GOLD 2022. Analysis of the patients’ clinical manifestations showed no differences in dyspnoea, BODEx index, CAT, COTE or FCI. There were also no differences when we compared the distribution by severity of dyspnoea (see Table 2).

**Table 2:** Lung function - Spirometry

Variables	No mutation n = 961	Mutation n = 144	All n = 1,105	p-value
FVC post-BD in litres, median (p25-p75)	2.37 (1.85–3.09)	2.51 (1.89–3.21)	2.4 (1.88–3.1)	0.259
FVC post-BD as percentage, median (p25-p75)	80 (67–95)	83 (67-99.5)	81 (67–95)	0.434
FEV1 post-BD in litres, median (p25-p75)	1.24 (0.93–1.72)	1.22 (0.96–1.70)	1.24 (0.94–1.71)	0.871
FEV1 post-BD as percentage, median (p25-p75)	58 (43–71)	58.5 (44.5–72)	58 (44–71)	0.862
FEV1/FVC ratio, median (p25-p75)	0.55 (0.45–0.64)	0.54 (0.44–0.62)	0.55 (0.44–0.63)	0.455
**COPD prediction scales**				
BODEx, mean ± SD	2.25 ± 1.75	2.28 ± 1.74	2.25 ± 1.75	0.847
CAT, mean ± SD	12.3 ± 7.6	11.9 ± 7.9	12.2 ± 7.6	0.624
COTE, mean ± SD	0.88 ± 1.61	0.87 ± 1.60	0.88 ± 1.61	0.907
FCI, mean ± SD	1.89 ± 1.09	1.8 ± 0.89	1.87 ± 1.06	0.398

COPD: chronic obstructive pulmonary disease; FVC: forced vital capacity; FEV1: forced expiratory volume in the first second; BD: bronchodilator; BODEx: BODE index; CAT: COPD Assessment Test; COTE: Comorbidity Test index; FCI: functional comorbidity index. Values as mean ± standard deviation or median (p25-p75) or n (%)

Of the 144 patients with mutations, the majority had the M/S mutation (78.5%), followed by M/Z (9.72%). Seventeen patients had at least one Z allele (11.8%), one patient had a ZZ mutation and two patients had null alleles (see Table 3).

**Table 3:** Subjects with mutation

Mutation found	n = 144
M/S, n (%)	113 (78.5)
M/Z, n (%)	14 (9.72)
S/S, n (%)	7 (4.87)
S/Z, n (%)	3 (2.08)
M/I, n (%)	3 (2.08)
M/Q0 mattawa, n (%)	1 (0.69)
S/I, n (%)	1 (0.69)
S/Plowell, n (%)	1 (0.69)
Z/Z, n (%)	1 (0.69)

Values presented as n (%)

In total, 23 patients had mutations associated with serum AAT deficiency (levels below 60 mg/dl), with a mean age of 73 years and 60.9% female. The FEV1/FVC ratio was 56%, with a mean CAT of 17, BODEx 2.5 and FEV1 of 1.22 litres (see Table 4).

**Table 4:** Characteristics of patients with AATD-related mutations

Variable	n = 23
Age, median (p25-p75)	73 (69.5–77.4)
Male, n (%)	14 (60.9)
**Lung function**	
FVC post-BD in litres, median (p25-p75)	2.47 (1.86–3.45)
FVC post-BD as percentage, median (p25-p75)	91 (64–105)
FEV1 post-BD in litres, median (p25-p75)	1.22 (0.97–2.02)
FEV1 post-BD as percentage, median (p25-p75)	62 (51–73)
FEV1/FVC, median (p25-p75)	0.56 (0.50–0.61)
**Prediction scales**	
CAT, mean ± SD	17.4 ± 11.1
BODEx, mean ± SD	2.5 ± 1.73

FVC: forced vital capacity; FEV1: forced expiratory volume in the first second; BD: bronchodilator; BODEx: BODE index; CAT: COPD Assessment Test. Values as mean ± standard deviation or median (p25-p75) or n (%)

**Fig. 1:**
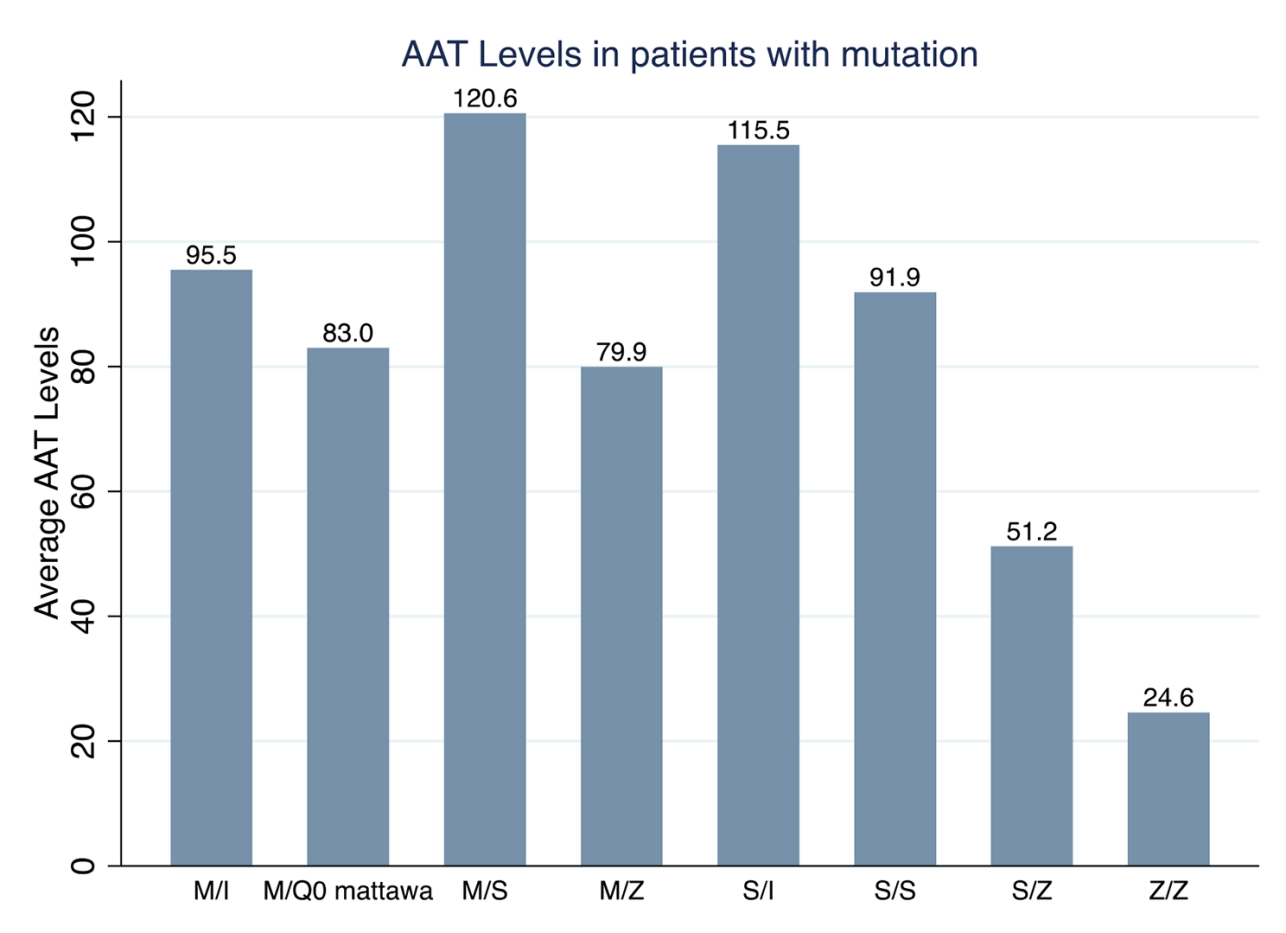
Alpha-1 antitrypsin levels in patients with mutation

In the patients with results available, serum AAT levels were below the treatable range in the case of SZ and ZZ mutations (see Fig. 1).

## Discussion

Having previously been regarded as rare, AATD is now considered to be a prevalent genetic disease. Current recommendations are for measurement of serum AAT levels in all patients with COPD, regardless of age and disease severity [12–15]. Although the alleles related to AATD make it a common genetic disease, its prevalence is low and in Colombia it is considered an unusual diagnosis.

AATD is associated with the presence of mutations in the SERPINA1 gene, which is located at the distal end of chromosome 14, consists of two alleles and is transmitted by simple Mendelian and autosomal codominant inheritance. Normal alleles, present in 85–90% of individuals, are called M [12, 16]. The most commonly occurring alleles in AATD are S and Z. In practice, most of the phenotypes found are combinations of the M, S, and Z alleles [17].

Screening for AATD can be performed on samples of oral mucosa, with an oral swab being a minimally invasive technique that avoids the need to take blood samples; moreover, this is the most recently developed diagnostic test. This technique offers greater DNA stability at temperature and humidity, and genotyping is performed by amplification using PCR techniques [8, 17].

Severe AATD is defined by the finding of serum AAT levels below 35% of the expected average value, or less than 57.2 mg/dl measured by nephelometry, or below 80 mg/dl if measured by radial immunodiffusion [12]. This condition is rare and is related to ZZ genotypes, occurring in 96% of ZZ homozygous patients and in 4% of SZ and MZ heterozygous patients [15].

In Colombia, it has been estimated that severe AATD could have a prevalence of 1 in 2,000 subjects with the SZ genotype and 1 in 24,000 subjects with severe genotype. However, these data were obtained by indirect theoretical estimates according to a 2012 publication [3]. This is the first study to date to provide data on the prevalence of AATD-related genes in patients with COPD in Colombia. Our prevalence data are similar to those published in previous studies in the United States and Europe, but higher than those reported in Latin American studies [6–9, 18].

A study carried out in Brazil published in 2016 reported AAT levels in 926 patients with COPD; they calculated an AATD prevalence of 2.8%, while the prevalence of the ZZ genotype, which corresponds to a severe AATD, was 0.8% [18]. Published in late 2019, the DAAT.AR study conducted in Argentina, which included 3,254 patients with a spirometry diagnosis of COPD, reported an adjusted prevalence of AATD in patients over the age of 40 of 0.83% [6].

The prevalence of the ZZ genotype in the population we studied was 0.69%, showing that severe AATD does exist in patients with COPD in Colombia and that all patients with COPD should be tested at least once in their lifetime for AATD, as recommended by the World Health Organization since 1999 [19], and supported by the American Thoracic Society (ATS)/European Respiratory Society (ERS) [12], the Canadian Thoracic Society [20] and the Sociedad Española de Neumología y Cirugía Torácica [Spanish Society of Respiratory Medicine and Thoracic Surgery] [21].

Comparison of the differences in clinical and paraclinical presentation between patients with and without mutations in our study called into question the traditional profile of suspecting deficiency only in COPD patients under the age of 40 and with lower lobe emphysema. The Caribbean and Pacific regions of Colombia have a population with a high percentage of people of African origin, while in the centre of the country, descendants of Spaniards and indigenous people predominate.

In this study, we compared the lung function and clinical manifestations (for example, dyspnea) in subjects with and without mutations. We did not find differences in spirometry parameters or in the subjects’ distribution on the dyspnea scale. This was probably because the severity of the disease at the time of diagnosis of COPD was similar between thegroups as they were both part of referral population at COPD centers in Colombia. It is worth mentioning that, in previous AATD studies, deficiency was analysed with AAT measurements in blood by nephelometry [8, 22, 23] and groups were classified by clinical risk of deficiency from these data. In our study, as genetic assessment is a fast, minimally invasive technique that can identify the mutations most commonly associated with AATD, we decided to evaluate the patients using this technique, meaning it was not necessary to measure serum levels in all patients [15, 24]. As serum AAT levels were not available in all patients, it is possible that new or severely deficient alleles were missed, so the actual prevalence could be underestimated in our study.

As we found a high proportion of patients with COPD to have a genetic component related to AATD, our results provide us with the evidence to raise awareness among doctors who care for patients with COPD here in Colombia, to remind them to request either serum AAT levels or oral mucosa swab genotyping, according to the availability at each centre. This information supports the early diagnosis of COPD, as described in other observational studies carried out in referral centres [25, 26].

From a Mendelian genetics point of view, we consider that this study does not deviate from Hardy-Weinberg principle because natural selection is not acting on the locus of AATD, there is no introduction of new alleles into our large population and individuals mate randomly with respect to this locus. We expect that genotype and allele frequencies in our population remain constant because they are in equilibrium.

The main strength of our study is that it is the first descriptive study carried out for this condition in Colombia including patients from seven centres distributed around the country. One of the limitations of the study was not having serum AAT levels available for all participants, because of selecting a different methodology for the study patients. Additionally, we did not include the patients’ chest computerised axial tomography results, which might have been helpful for enabling a correlation between radiology and mutations. Another limitation of the study is that in the genotyping test, the MM genotype was identified by excluding other mutations.

The design of our study was cross-sectional and may have selection and misclassification bias. To reduce selection bias, we enrolled subjects using a non-random sampling technique of consecutive cases. To avoid misclassification, we collected subjects who met the eligibility criteria for the diagnosis of COPD including subjects from 18 years of age. Since we included COPD subjects with long-standing diagnosis, the results obtained may not represent patients with more severe disease, as a potential survival bias.

## Conclusions

To our knowledge, this is the first study to date to provide data on the prevalence of AATD-related genes in patients with COPD in Colombia and can serve as the first step for initiating new studies exploring AATD in COPD subjects. Mutations were documented in 13.01% of patients with COPD in Colombia and 2.07% were AATD-related, showing that there is a significant number of underdiagnosed patients. It is therefore necessary to raise awareness of COPD-related AATD, and these results reinforce the need for genetic study and/or measurement of serum AAT levels in patients diagnosed with COPD here in Colombia.

## Data Availability

All data supporting the findings of this study are available in the article. Additional information is available from the corresponding author upon reasonable request.
